# Malnutrition affects cholesterol paradox in coronary artery disease: a 41,229 Chinese cohort study

**DOI:** 10.1186/s12944-021-01460-6

**Published:** 2021-04-19

**Authors:** Bo Wang, Jin Liu, Shiqun Chen, Ming Ying, Guanzhong Chen, Liwei Liu, Zhubin Lun, Huanqiang Li, Haozhang Huang, Qiang Li, Yaren Yu, Mengfei Lin, Wen Wei, Zhidong Huang, Yongquan Yang, Jiyan Chen, Ning Tan, Yong Liu

**Affiliations:** 1 Department of Cardiology, Guangdong Provincial Key Laboratory of Coronary Heart Disease Prevention, Guangdong Cardiovascular Institute, Guangdong Provincial People’s Hospital, Guangdong Academy of Medical Sciences, Guangzhou, 510080 China; 2 Guangdong Provincial People’s Hospital, School of Medicine, South China University of Technology, Guangzhou, 510100 China; 3 The Second School of Clinical Medicine, Southern Medical University, Guangzhou, 510515 China; 4 The First School of Clinical Medicine, Guangdong Medical University, Zhanjiang, 523808 China; 5 Department of Cardiology, The First People’s Hospital of Foshan, No. 81 of Lingnan Road, Foshan, 528000 China; 6 Maoming People’s Hospital, Maoming, 525000 China; 7 Department of Cardiology, Longyan First Hospital Affiliated with Fujian Medical University, Longyan, 364000 China

**Keywords:** Low-density lipoprotein cholesterol, Coronary artery disease, Malnutrition, Long-term all-cause mortality

## Abstract

**Background:**

Several studies have found that a low baseline low -density lipoprotein cholesterol (LDL-C) concentration was associated with poor prognosis in patients with acute coronary syndrome (ACS), which is called the “cholesterol paradox”. Low LDL-C concentration may reflect underlying malnutrition, which was strongly associated with increased mortality. The aim of this study was to investigate the cholesterol paradox in patients with CAD and the effects of malnutrition.

**Method:**

A total of 41,229 CAD patients admitted to Guangdong Provincial People’s Hospital in China were included in this study from January 2007 to December 2018 and divided into two groups (LDL-C < 1.8 mmol/L, *n* = 4863; LDL-C ≥ 1.8 mmol/L, *n* = 36,366). The Kaplan-Meier method and Cox regression analyses were used to assess the association between LDL-C levels and long-term all-cause mortality and the effect of malnutrition.

**Result:**

In this real-world cohort (mean age 62.9 years; 74.9% male), there were 5257 cases of all-cause death during a median follow-up of 5.20 years [interquartile range (IQR): 3.05–7.78 years]. Kaplan–Meier analysis showed that low LDL-C levels were associated with a worse prognosis. After adjusting for baseline confounders (e.g., age, sex and comorbidities, etc.), multivariate Cox regression analysis revealed that a low LDL-C level (< 1.8 mmol/L) was not significantly associated with all-cause mortality (adjusted HR, 1.04; 95% CI, 0.96–1.24). After adjustment for nutritional status, the risk of all-cause mortality in patients with low LDL-C levels decreased (adjusted HR, 0.90; 95% CI, 0.83–0.98). In the final multivariate Cox model, a low LDL-C level was related to better prognosis (adjusted HR, 0.91; 95% CI, 0.84–0.99).

**Conclusion:**

This study demonstrated that the cholesterol paradox existed in CAD patients but disappeared after accounting for the effects of malnutrition.

## Introduction

Increased serum low-density lipoprotein cholesterol (LDL-C) constitutes a major risk factor of poor prognosis of coronary artery disease (CAD) [1, 2]. Interestingly, findings from recent studies demonstrated a paradoxical association of low LDL-C levels with poor prognosis in patients with acute coronary syndrome (ACS), which is so-called the “cholesterol paradox” [3–7]. LDL-C concentration was highly correlated with total cholesterol level, which is an indicator of nutritional status [8, 9]. Meanwhile, malnutrition is common in CAD patients and strongly correlates with increased long-term mortality [10, 11]. Thus, low LDL-C levels may represent underlying malnutrition, which was related to the paradox.

Several factors may account for the “cholesterol paradox”, including a higher proportion of elderly patients and a higher proportion of baseline comorbidities [4, 5, 7]. However, current studies did not address the impact of malnutrition on the cholesterol paradox.

Therefore, this study sought to inquire into the association of baseline low LDL-C concentration with long-term all-cause mortality in patients complicated with CAD after considering the effects of malnutrition.

## Methods

### Study design and participants

**Fig. 1:**
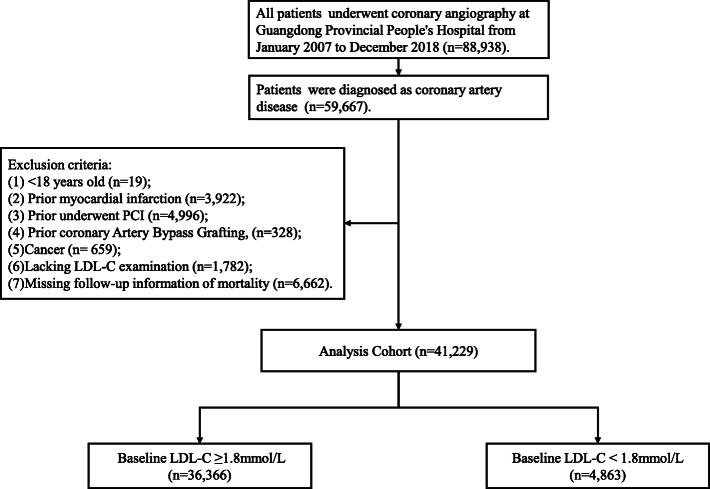
Study flow chart

This retrospective observational study was conducted in Guangdong Provincial People’s Hospital, China (Clinicaltrials.gov NCT04407936). From January 2007 to December 2018, all hospitalised patients who underwent cardiac catheterisation were included. During this period, 88,938 patients undergoing coronary angiography (CAG), and 59,667 patients were diagnosed with CAD. The exclusion criteria included < 18 years old (*n* = 19), prior myocardial infarction (*n* = 3922), prior PCI (*n* = 4996), prior coronary artery bypass grafting (*n* = 328), cancer (*n* = 659), missing LDL-C examination (*n* = 1782) and missing follow-up information on mortality (*n* = 6662). Finally, the study included 41,229 patients (Fig. 1). Study protocol was approved by the Ethics Committee of Guangdong Provincial People’s Hospital (No. GDREC2019555H[R1]), which complied with the Declaration of Helsinki.

### Procedures

Clinical data for each patient with CAD was extracted from the electronic Clinical Management System. Baseline data were collected on demographic characteristics, coexisting conditions, laboratory tests, and medication at discharge. In all patients, lipid levels were measured in overnight fasting blood samples. Other blood samples were collected at admission or before coronary angiography (CAG) and percutaneous coronary intervention (PCI). CAG/PCI was performed complying with the standard clinical practice guidelines [12–14]. Senior cardiologists were responsible for data quality control and periodical data verification. Follow-up information was retrieved from the Guangdong Public Security System.

### Clinical outcome and definition

The primary endpoint was long-term all-cause mortality. CAD was diagnosed by CAG and was depicted as 50% stenosis of at least one coronary artery. CAD was also determined according to the International Statistical Classification of Diseases and Related Health Problems 10th Revision (ICD-10). In addition, comorbidities included acute myocardial infarction (AMI), diabetes mellitus, hypertension, congestive heart failure (CHF), chronic kidney disease (CKD) etc. The definition of CHF was Killip class ≥2 or New York Heart Association class ≥3 [15]. The value of estimated glomerular filtration rate (eGFR) was calculated by MDRD formula, and CKD was recognized as eGFR ≤60 mL/min/1.73 m^2^ [16–18]. Anaemia was defined as haematocrit < 39% for men and haematocrit < 36% for women, in accordance with World Health Organization criteria [19]. Controlling Nutritional Status (CONUT) score is a screening tool of nutritional status for hospitalised patients and was applied in this study [8]. It was calculated by cholesterol concentration, plasma albumin level and total lymphocyte count. Different scores represent different nutrition statuses (0–1 represents normal; 2–4 represents mild malnutrition; 5–8 represents moderate malnutrition; 9–12 represents severe malnutrition).

### Statistical analysis

This study divided patients into two groups according to the concentration of LDL-C: a high LDL-C group (≥1.8 mmol/L) and a low LDL-C group (< 1.8 mmol/L). The normality test of continuous variables was performed by visual inspection (histograms and Q-Q plots). Descriptive statistics for categorical variables, continuous variables with normal distribution and abnormal distribution are expressed as numbers (percentages), mean [standard deviation (SD)] and median (interquartile range [IQR]), respectively. Independent sample Student’s t-test was conducted for continuous variables with a normal distribution. Pearson chi-square test was performed for categorical variables. The prognosis was analysed by the Kaplan-Meier method, and the probability of outcomes in the two groups was analysed by the survival curve. For comparison of survival differences between the two groups, this study used the log-rank test. Unadjusted and adjusted Cox proportional hazard models were used to investigate the relationship between baseline LDL-C level and long-term all-cause mortality by calculating hazard ratio. Baseline variables that were considered clinically relevant were included in the multivariate Cox regression model. In this study, the variables included were carefully selected in accordance with available events number given to ensure the simplicity of final model. The following four models were sequentially constructed with or without adjustment for covariates: 1) unadjusted; 2) adjusted age ≥ 75 years, sex and comorbidities including AMI, diabetes mellitus, hypertension, atrial fibrillation, COPD, CHF, CKD and anaemia; 3) adjusted nutritional status (CONUT score); and 4) adjusted for all covariates. All data analyses were carried out using R software, version 3.6.3 (R Foundation for Statistical Computing). All *P* values < 0.05 were set to statistically significant.

## Results

### Clinical characteristics

**Fig. 2:**
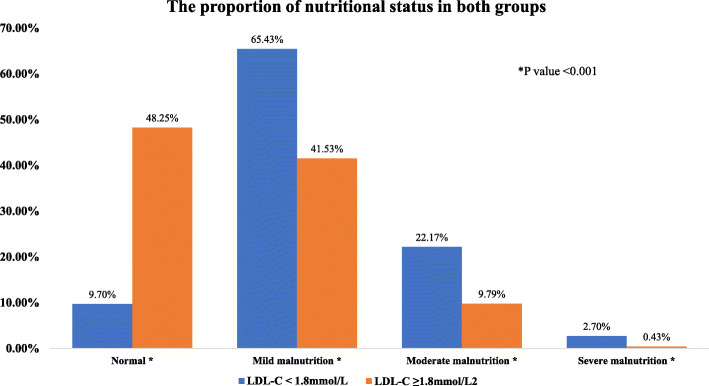
The proportion of different nutritional statuses in the LDL-C < 1.8 mmol/L group and LDL-C ≥ 1.8 mmol/L group

Forty-one thousand two hundred twenty-nine CAD patients were included in the final analysis. Table 1 exhibited the baseline clinical characteristics for enrolled patients. As expected, patients with LDL cholesterol level < 1.8 mmol/L were older and more likely to develop comorbidities than those with LDL cholesterol concentration ≥ 1.8 mmol/L. Notably, prevalence of malnutrition was particularly high in the low baseline LDL-C concentration group (Fig. 2).

**Table 1:** Baseline characteristics

Characteristic^a^	Overall (*N* = 41,229)	LDL-C < 1.8 mmol/L (*N* = 4863)	LDL-C ≥ 1.8 mmol/L (*N* = 36,366)	*P* value
**Demographic characteristics**				
Age, years	62.9 (10.6)	64.4 (10.8)	62.8 (10.6)	< 0.001
Age ≥ 75 years, n (%)	6036 (14.6)	929 (19.1)	5107 (14.0)	< 0.001
Male, n (%)	30,897 (74.9)	3725 (76.6)	27,172 (74.7)	0.005
**Coexisting conditions**				
PCI, n (%)	30,415 (73.8)	3433 (70.6)	26,982 (74.2)	< 0.001
AMI, n (%)	9182 (22.3)	805 (16.6)	8377 (23.0)	< 0.001
CHF, n (%)	3902 (9.5)	409 (8.4)	3493 (9.6)	0.008
Hypertension, n (%)	23,217 (56.3)	3020 (62.1)	20,197 (55.5)	< 0.001
Diabetes mellitus, n (%)	11,097 (26.9)	1623 (33.4)	9474 (26.1)	< 0.001
CKD, n (%)	8635 (21.9)	1154 (24.8)	7481 (21.5)	< 0.001
Atrial fibrillation, n (%)	939 (2.3)	133 (2.7)	806 (2.2)	0.026
COPD, n (%)	348 (0.8)	54 (1.1)	294 (0.8)	0.038
Stroke, n (%)	2340 (5.7)	347 (7.1)	1993 (5.5)	< 0.001
Anaemia, n (%)	12,922 (32.3)	2079 (44.0)	10,843 (30.7)	< 0.001
**Laboratory examination**				
Haematocrit	0.40 (0.05)	0.38 (0.05)	0.40 (0.05)	< 0.001
Lymphocyte, 10^9^/L	1.94 (0.71)	1.95 (0.71)	1.83 (0.69)	< 0.001
Total cholesterol, mmol/L	4.60 (1.21)	4.78 (1.13)	3.24 (0.91)	< 0.001
HDL-C, mmol/L	1.00 (0.26)	1.01 (0.26)	0.93 (0.30)	< 0.001
LDL-C, mmol/L	2.85 (0.98)	1.47 (0.26)	3.04 (0.88)	< 0.001
Triglyceride, mmol/L	1.67 (1.23)	1.67 (1.09)	1.68 (1.96)	0.304
Albumin, g/L	36.29 (4.25)	36.36 (4.22)	35.74 (4.44)	< 0.001
**Medicine**				
RASi, n (%)	20,082 (49.6)	2199 (46.5)	17,883 (50.00)	< 0.001
β-blocker, n (%)	32,652 (80.6)	3776 (79.8)	28,876 (80.7)	0.131
Statins, n (%)	38,300 (94.6)	4396 (92.9)	33,904 (94.8)	< 0.001
**Events**				
All-cause death, n (%)	5257 (12.8)	778 (16.0)	4479 (12.3)	< 0.001

^a^Data are presented as the mean value (standard deviation), median [interquartile range] or number of participants (percentage)

Abbreviations: *LDL-C* Low-density lipoprotein cholesterol, *PCI* Percutaneous coronary intervention, *AMI* Acute myocardial infarction, *CHF* Congestive heart failure, *CKD* Chronic kidney injury, *COPD* Chronic obstructive pulmonary disease, *HDL-C* High-density lipoprotein cholesterol, *RASi* Renin angiotensin system inhibitor

### Primary outcomes

**Fig. 3:**
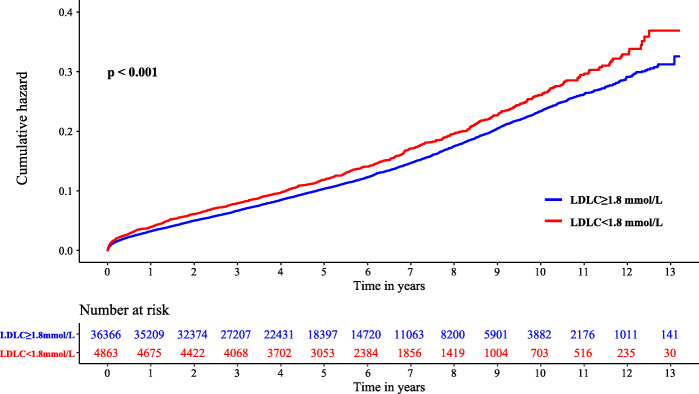
Cumulative incidence of all-cause death for the LDL-C < 1.8 mmol/L group vs. the LDL-C ≥ 1.8 mmol/L group in CAD patients

**Fig. 4:**
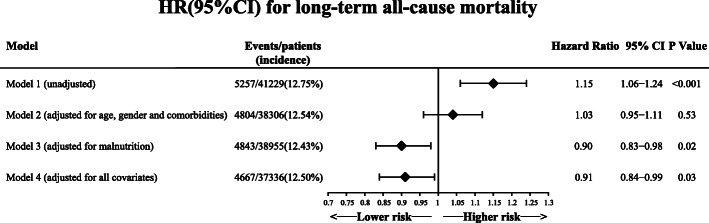
Unadjusted and adjusted HRs and 95% CIs for the primary end point (long-term all-cause mortality) of the LDL-C < 1.8 mmol/L group vs. LDL-C ≥ 1.8 mmol/L group in CAD patients. Model 1: Unadjusted model. Model 2: Adjusted for age ≥ 75 years, sex and comorbidities including AMI, diabetes mellitus, hypertension, atrial fibrillation, COPD, CHF, CKD and anaemia. Model 3: Adjusted for malnutrition. Model 4: Adjusted for all covariates: age ≥ 75 years, sex and comorbidities including AMI, diabetes mellitus, hypertension, atrial fibrillation, COPD, CHF, CKD, anaemia and malnutrition

Over a median follow-up period of 5.20 years (interquartile range, 3.05–7.78 years), the rate of all-cause mortality was 12.8% (*n* = 5257). Kaplan-Meier analysis revealed that prognosis in patients with LDL-C concentration < 1.8 mmol/L was worse (Fig. 3). Confounders were eliminated using Cox regression analysis to determine the difference in prognosis between patients with low LDL-C level (< 1.8 mmol/L) and patients with high LDL-C level (≥ 1.8 mmol/L) (Fig. 4). With adjustment for age, sex and comorbidities (model 2), patients in the low LDL-C group (< 1.8 mmol/L) had a nonsignificant difference in long-term all-cause mortality (adjusted HR: 1.04, 95% CI: 0.96–1.12, Fig. 4). However, after adjustment for nutritional status (model 3), patients with low LDL-C concentration (< 1.8 mmol/L) had lower risks of long-term all-cause mortality (adjusted HR: 0.90, 95% CI: 0.83 to 0.98, Fig. 4). After adjustment for full covariates (model 4), patients with low LDL-C level (< 1.8 mmol/L) had a 9% reduced risk of long-term all-cause death compared with those in high LDL-C group (> 1.8 mmol/L) (adjusted HR: 0.91; 95% CI: 0.84 to 0.99, Fig. 4). Malnutrition was the most important confounder in the association of low LDL-C levels with clinical outcomes (Fig. 4).

## Discussion

To our knowledge, few studies have investigated the correlation between baseline LDL-C concentration and long-term all-cause mortality in CAD patients. This study found that the worse prognosis of patients across the low LDL-C group (< 1.8 mmol/L) is mainly mediated by their higher prevalence of malnutrition. This study demonstrated that the cholesterol paradox also existed among people with CAD. After considering the marked differences in age, sex and proportion of comorbidities, the differences were not significant between patients with different LDL-C concentration. After adjustment for malnutrition, CAD patients owing low baseline serum LDL-C concentrations showed a low risk of long-term all-cause mortality.

This study showed a negative correlation between baseline plasma LDL-C level and long-term outcomes in unadjusted analysis. The cholesterol paradox was also observed in other studies involving subgroups of CAD patients (e.g., ACS) [3–7], highlighting the impact of baseline confounders. Cho et al. found that among patients complicated with acute myocardial infarction (AMI), a lower baseline LDL-C concentration (< 1.8 mmol/L) was correlated with higher 1-year mortality before adjusting for baseline confounders [3]. In that study, the age and rate of comorbidities decreased as LDL-C increased. According to the Cox regression results, LDL-C level was not independently related to 1-year mortality with adjustment for baseline confounders. Wang et al. demonstrated that a higher LDL-C level at admission was correlated with better in-hospital survival in patients with ACS in the unadjusted analysis [4]. Similarly, the results suggest that the cholesterol paradox may be due to other clinical characteristics. Reddy et al., Al-Mallah et al. and Nakahashi et al. demonstrated that a lower baseline LDL-C concentration was associated with higher mortality before and after adjusting for baseline confounders [5–7]. All five studies attempted to explore the mechanisms behind the paradox by taking baseline confounding factors into account, which included demographic information and comorbidities. There are noteworthy similarities between our study and the five previous studies. First, these study groups were all patients with CAD. Second, the LDL-C levels were all the baseline values collected at admission. Third, after adjusting for baseline confounders (age, sex, comorbidity, etc.), lower baseline LDL-C concentration was not associated with decreased long-term mortality.

However, the influence of nutritional status was not considered in depth. According to available data from the five studies, patients with lower LDL-C also had lower plasma albumin and total cholesterol concentrations, which may reflect underlying malnutrition status. The CONUT score is an effective scale for assessment in early stage and ongoing monitoring of hospital nutrition status [8]. In our study, the prevalence of malnutrition was 56.3, 51.8 and 90.3% in the overall population, high LDL-C group (≥ 1.8 mmol/L) and low LDL-C group (< 1.8 mmol/L), respectively. The malnutrition rate in patients with low LDL-C concentrations was much higher than that in patients with high LDL-C concentrations. Other studies also found this situation, highlighting the importance of nutritional status [10, 11]. Roubín et al.’s findings showed that in patients with ACS, 38.5, 10.4 and 0.9% of patients were mildly, moderately and severely malnourished according to the CONUT score, respectively [10]. Roubín et al. and Wada et al. found that nutritional status evaluated by the CONUT score was significantly correlated with the long-term clinical outcome for CAD patients [10, 11].

There may be several possible explanations for the cholesterol paradox. First, a plausible explanation for the absence of a positive correlation between baseline LDL-C and long-term all-cause mortality before the adjustment is that these patients have a higher proportion of elderly people (≥ 75 years) and comorbidities according to baseline characteristics, which was associated with a worse prognosis. Patients who were in low LDL cholesterol levels (< 1.8 mmol/L) had a significantly higher prevalence than those with LDL cholesterol ≥1.8 mmol/L in patients aged ≥75 years (19.1% vs. 14.0%), anaemia (43.2% vs. 30.3%), diabetes mellitus (33.4% vs. 26.1%) and atrial fibrillation (2.7% vs. 2.2%). Additionally, increased long-term mortality may result from basic diseases to some extent. Smokers have a higher survival rate after AMI (the smoker paradox) [20]. The smoker paradox can be explained as follows: smokers are younger and have fewer cardiovascular risk factors than non-smokers. In addition, some previous studies have also reported that patients with higher BMI have a better prognosis after PCI (the obesity paradox) [21, 22]. Studies have demonstrated that baseline confounders associated with prognosis may contribute to the obesity paradox [23–25]. In our study, multivariate Cox regression analysis also revealed that baseline LDL-C concentration was no longer independently relevant to mortality after adjusting for age, sex and comorbidities. Second, patients with lower LDL-C levels were at poorer nutritional status. Malnutrition, in particular, significantly affected prognosis. Compared with the unadjusted model, the linkage between low LDL-C levels and prognosis changed from negative to positive after adjustment for malnutrition. A previous study found that low level of total cholesterol was a biological marker of cancer, concurrent cachexia, other chronic diseases and malnutrition and has been shown to have an adverse effect on survival [26]. Evidence is emerging that cholesterol is related to the regulation of immune cell function by improving their antitumour activity and activating immune signalling, which may provide novel insights into the action of cholesterol in cancer development [27–29]. Furthermore, according to some other articles, low LDL levels have also been reported to be associated with decreased cognitive function, depression and mood disorders [30–32]. This may cause an increased risk of long-term all-cause death. Malnutrition also often represents secondary immune dysfunction [33]. If the development of malnutrition cannot be recognized or predicted, unnecessary malnutrition and susceptibility to infection may occur, thereby increasing morbidity and mortality. Therefore, the cholesterol paradox in CAD patients may be mainly attributed to malnutrition.

All these findings strongly support that physicians need to incorporate malnutrition identification into their daily practice. This can improve risk stratification and guide follow-up interventions for secondary prevention. The effect of malnutrition should be considered when LDL-C is used to assess the risk of poor prognosis in CAD patients. Clinicians should not let down their guard when they meet CAD patients with low LDL-C levels. Screening for malnutrition in CAD patients may help discern population who are at high risk of poor prognosis, and these patients may benefit from pertinent nutritional supplements and secondary prevention programmes which can improve their prognosis.

### Study strength and limitations

There were several strengths to this study. First, the study was conducted at Guangdong Provincial People’s Hospital, the largest cardiovascular centre in South China, and the sample size was substantial. Second, this was the first study to consider the effect of malnutrition on the cholesterol paradox. Based on previous studies, this study comprehensively considered age, sex, comorbidities and nutritional status and found that the cholesterol paradox no longer existed after taking nutritional status into account. Third, the current study suggested that the nutritional status of patients should be evaluated to make the right decision before intensive lipid-lowering therapy is initiated. However, there were some limitations to this analysis. First, this study was a retrospective single-centre study. Second, only value of LDL-C at admission was used in this study, making it difficult to assess the impact of changes in LDL-C concentration at follow-up on clinical outcomes. Therefore, the focus of this study was the clinical significance for baseline LDL-C concentration on prognosis among CAD patients. Third, there was limited data on the included patients, without information about body weight, BMI, waist circumference or adiposity, which might help us assess nutritional status comprehensively. However, we chose the CONUT score based on three laboratory indicators as a nutritional status assessment tool. This may also objectively evaluate the nutritional status of the patient. Finally, the type and doses of statins were not specified in our database. However, based on baseline data, more than 90% of patients were treated with statins, and the inclusion of statin therapy in the multivariate analysis did not change the results.

## Conclusion

The cholesterol paradox persists in CAD patients. However, after removing the effect of malnutrition, a low baseline LDL-C level (< 1.8 mmol/L) was correlated with a decreased risk of long-term all-cause death. Our findings indicate that CAD patients’ low LDL-C levels on admission did not mean a low risk of long-term all-cause mortality, and low LDL-C levels should be considered because CAD patients probably have underlying malnutrition. It is necessary to evaluate the nutrition status of patients with CAD. Nutrition status should not be ignored when patients receive lipid-lowering therapy.

## Data Availability

Not applicable at this stage. The datasets analysed during the current study will be available from the corresponding author on a reasonable request when the study is finished.

## Abbreviations

LDL-C: Low-density lipoprotein cholesterol
CAD: Coronary artery disease
CAG: Coronary angiography
PCI: Percutaneous coronary intervention
AMI: Acute myocardial infarction
CHF: Congestive heart failure
CKD: Chronic kidney disease
COPD: Chronic obstructive pulmonary disease
HR: Hazard ratio
CI: Confidence interval
